# Nonelectroactive *clostridium* obtains extracellular electron transfer-capability after forming chimera with *Geobacter*

**DOI:** 10.1093/ismeco/ycae058

**Published:** 2024-04-25

**Authors:** Xing Liu, Yin Ye, Naiming Yang, Chen Cheng, Christopher Rensing, Chao Jin, Kenneth H Nealson, Shungui Zhou

**Affiliations:** Fujian Provincial Key Laboratory of Soil Environmental Health and Regulation, College of Resources and Environment, Fujian Agriculture and Forestry University, Fuzhou, Fujian 350002, China; Fujian Provincial Key Laboratory of Soil Environmental Health and Regulation, College of Resources and Environment, Fujian Agriculture and Forestry University, Fuzhou, Fujian 350002, China; Fujian Provincial Key Laboratory of Soil Environmental Health and Regulation, College of Resources and Environment, Fujian Agriculture and Forestry University, Fuzhou, Fujian 350002, China; Fujian Provincial Key Laboratory of Soil Environmental Health and Regulation, College of Resources and Environment, Fujian Agriculture and Forestry University, Fuzhou, Fujian 350002, China; Fujian Provincial Key Laboratory of Soil Environmental Health and Regulation, College of Resources and Environment, Fujian Agriculture and Forestry University, Fuzhou, Fujian 350002, China; School of Environmental Science and Engineering, Sun Yat-Sen University, Guangzhou, Guangdong 510006, China; Department of Earth Science & Biological Sciences, University of Southern California, Los Angeles, CA 91030, United States; Fujian Provincial Key Laboratory of Soil Environmental Health and Regulation, College of Resources and Environment, Fujian Agriculture and Forestry University, Fuzhou, Fujian 350002, China

**Keywords:** Gram-positive bacteria, energetic coupling, extracellular electron transfer, interspecies electron transfer, energy metabolism, electroactive bacteria, interspecies mutualism

## Abstract

Extracellular electron transfer (EET) of microorganisms is a major driver of the microbial growth and metabolism, including reactions involved in the cycling of C, N, and Fe in anaerobic environments such as soils and sediments. Understanding the mechanisms of EET, as well as knowing which organisms are EET-capable (or can become so) is fundamental to electromicrobiology and geomicrobiology. In general, Gram-positive bacteria very seldomly perform EET due to their thick non-conductive cell wall. Here, we report that a Gram-positive *Clostridium intestinale* (C.i) attained EET-capability for ethanol metabolism only after forming chimera with electroactive *Geobacter sulfurreducens* (G.s). Mechanism analyses demonstrated that the EET was possible after the cell fusion of the two species was achieved. Under these conditions, the ethanol metabolism pathway of C.i was integrated by the EET pathway of G.s, by which achieved the oxidation of ethanol for the subsequent reduction of extracellular electron acceptors in the coculture. Our study displays a new approach to perform EET for Gram-positive bacteria via recruiting the EET pathway of an electroactive bacterium, which suggests a previously unanticipated prevalence of EET in the microbial world. These findings also provide new perspectives to understand the energetic coupling between bacterial species and the ecology of interspecies mutualisms.

## Graphical Abstract

**Graphical Abstract:**
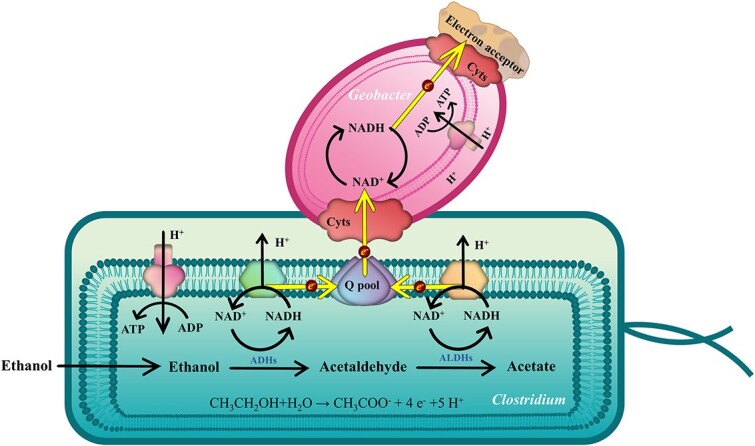

## Introduction

Extracellular electron transfer (EET) is the process whereby some microorganisms exchange electrons with the outside environment and is the prominent characteristic that defines an electroactive (EET-capable) microorganism [1]. EET-capable microorganisms were first noted and reported by their ability to perform anaerobic respiration using insoluble metal oxides as electron acceptors [2, 3]. They have since been shown to utilize anodic electrodes [4], and even other bacteria [5, 6] as electron acceptors. Other studies have revealed the ability to carry out chemoautotrophic growth by taking up electrons from low valence state iron as well as from other bacterial electron donors [7]. Thus, EET can facilitate the electron transfer between different species, contributing to such key ecological and environmental processes as anaerobic methane generation and oxidation, anaerobic ammonia oxidation, anaerobic photosynthesis, and anaerobic dark CO_2_ fixation [8–12]. Moreover, it also lays the foundation for the development of bioelectrochemical systems that exploit the electron transfer between microorganisms and electrodes for current generation or value-added chemical production [13, 14]. Clearly, the study of EET has greatly expanded our understanding and appreciation of the interaction(s) between microorganisms and their outside world, and has garnered considerable attention in the fields of geomicrobiology and electromicrobiology, especially in anoxic ecosystems such as those found in sediments, the deep subsurface, and animal gut microbiomes [7, 14].

Our understanding of the mechanisms that enable EET rests mainly on studies of Gram-negative bacteria [15, 16]. In general, two mechanisms of direct electron transfer (DET) and mediated electron transfer (MET) have been shown. Briefly, to achieve DET, bacteria either express transmembrane porin-cytochrome protein complex to transfer electrons to the directly physically contacted electron acceptors, or express conductive nanowires to perform long-range DET to reduce remote electron acceptors [17–19]. In contrast, for MET, redox active mediators either secreted by bacterial cells or preexisting in the environment have been shown to act at high concentrations as electron shuttles to mediate electron transfer between bacterial cells and extracellular electron acceptors [20–24]. In contrast, Gram-positive bacteria contain thick non-conductive cell walls (20–80 nm) that are composed of peptidoglycan, teichoic acids, and sometimes are covered with a protective glycoprotein S layer [25, 26]. Therefore, Gram-positive bacteria have generally been thought to be non-electroactive with only a few species having been reported to perform EET. For example, it was reported that *Lactococcus lactis* excreted the redox mediator quinone to enable anode reduction [27], and *Listeria monocytogenes* was shown to express flavoproteins that cooperated with flavin shuttles to transfer electrons extracellularly [28]. *Lysinibacillus varians* utilized a conductive nanowire to achieve EET [29], while *Thermincola potens* and *Carboxydothermus ferrireducens* contained multiple c-type cytochrome encoding genes and were proposed to express extracellular cytochromes to aid in the reduction of extracellular electron acceptors [30, 31].

*Clostridium* is a widely distributed Gram-positive genus in the phylum firmicutes. It is a strict fermentative anaerobe that typically produces hydrogen, organic solvents, and/or organic acids as fermentation by-products [32, 33]*. Clostridia* have been found as predominant genera in mixed species electroactive biofilms performing anode reduction [34, 35]. However, only a few *Clostridia* species have been reported to be electroactive with the EET mechanism(s) remaining unknown [36–38], and most were believed to only ferment the complex organics for the production of electron donors benefitting the cohabitating electroactive bacteria [39, 40].

**Figure 1:**
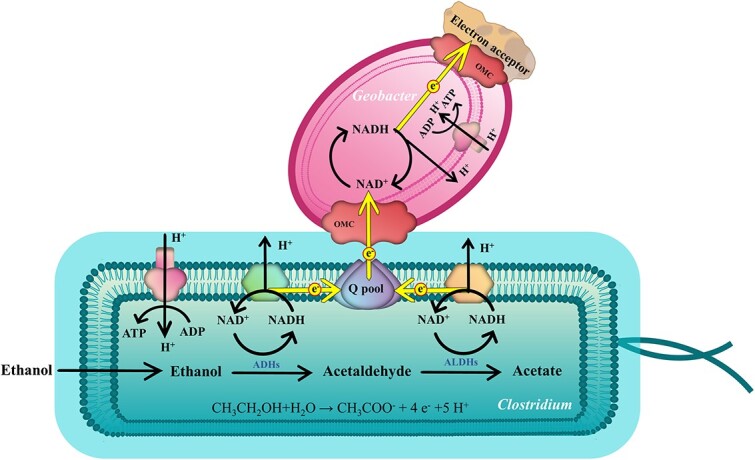
The proposed EET mechanism of C.i when cocultured with G.s; C.i catabolized ethanol for the generation of acetaldehyde by ethanol dehydrogenases (ADHs) and further oxidized to acetate by acetaldehyde dehydrogenases (ALDHs), and electrons were released and accumulated as NADH during this process simultaneously; in the coculture, G.s fused its cell with C.i by which the cytochrome-based EET pathway of G.s integrated with the energy metabolic pathway of C.i and thereafter discharged the C.i cell for NADH oxidation; such process contributed to the formation of proton motive force for the energy generation of C.i; at the same time, the electrons transferred into G.s and finally used for the reduction of extracellular electron acceptors with the energy generated in G.s concomitantly; here, the two species formed an energetic coupling that enabled both ethanol oxidation and extracellular electron acceptor reduction synchronously; Cyts is cytochromes.

We report here that non-electroactive *Clostridium intestinale* (C.i) acquired EET ability for ethanol catabolism when cocultured with electroactive *Geobacter sulfurreducens* (G.s) (Fig. 1). C.i was not able to catabolize ethanol since the produced electrons or energy were accumulated as NADH in the cell which results from the lack of a mechanism to discharge from the cell for NAD^+^ replenishment. When grown with G.s, these two species formed an intimate connection via cell fusion by which the cytochrome-based EET pathway of G.s was integrated with the energy metabolic pathway of C.i, providing an EET pathway to C.i for NADH oxidation and energy generation. Meanwhile, G.s transferred the electrons to extracellular electron acceptors for energy production supporting the growth of itself. In this manner, the ethanol oxidation by C.i was driven by and synchronized with the extracellular electron acceptor reduction of G.s. That is, C.i and G.s established energetic coupling (Fig. 1).

## Materials and methods

### Bacterial strains and culture conditions

C.i strain Lx1 (Genebank accession number: CP059378.1) and G.s strain PCA (ATCC 51573) were obtained from our laboratory culture collection, as were G.s mutant strains G.s-Δ*fdnG*Δ*hybL* (both GSU0777 and GSU0785 deletion), G.s*-*Δ*gltA* (GSU1106 deletion), G.s*-*Δ*omcS* (GSU2504 deletion), and G.s*-*Δ*omcBEST* (quadruple genes of GSU2737, GSU0618, GSU2504, and GSU2503 deletion) [41, 42]. C.i was routinely cultured in modified DSMZ medium 135 omitting sodium resazurin, and G.s strains were grown in bicarbonate-based medium containing 20 mM acetate as electron donor and 40 mM fumarate as electron acceptor [43]. When performing the Fe(III) reduction tests, a bicarbonate-based medium supplied with 20 mM ethanol as electron donor and either 56 mM ferric citrate or 100 mM poorly crystalline Fe(III) oxide as the electron acceptor was used to culture cells [43]. All cultures were routinely incubated at 30°C under anaerobic conditions (80:20 N_2_:CO_2_) as previously described [42].

### Bioelectrochemical system construction and operation

A three-electrode H-shaped anaerobic bioelectrochemical system was constructed (Fig. S1). It consisted of an anode chamber and a cathode chamber with a work volume of 22 ml for each. The two chambers were separated by a proton exchange membrane (Nafion N-117, Thermo Scientific Alfa Aesar, Waltham, MA). The anodic electrode was an indium tin oxide-coated glass plate (Sunyo, Suzhou, China), and the cathodic electrode was made of polished graphite carbon plate (HP Graphite Ltd, Handan, China). The salt bridge filled with saturated KCl was mounted with a saturated calomel electrode to act as the reference electrode [44]. The electrolyte was the anaerobic fresh water medium (FWNN) except that a final concentration of 20 mM ethanol or 20 mM acetate was supplied in the anolyte as electron donor [42]. Both chambers were kept under strictly anaerobic conditions (80:20 N_2_:CO_2_). To inoculate, cells were washed with FWNN beforehand. When running electrochemical tests, a constant voltage of 0.3 V (versus Hg/HgCl sat. KCl) was applied to the working electrode and the current was recorded simultaneously using a potentiostat (CH Instrument, Inc., Shanghai, China). Cyclic voltammetry was performed *in situ* under non-turnover conditions using CHI660E (CH Instrument) by scanning the electrode from −0.6 to 0.3 V with a scanning rate of 10 mV s^−1^. All tests were performed at 30°C.

### Coulombic efficiency calculation

Coulombic efficiency (*C_E_*; %) was determined by integrating the current over time:


$$ {C}_E=\frac{M_s\int_0^{t_b} Idt}{Fb{V}_{An}\varDelta c}\ast 100\% $$

where *M_S_* is the molecular weight of ethanol, *t_b_* is the total running of time, *I* is the current, *F* is the Faraday’s constant, *b* is the number of moles electrons generated per mole of ethanol, *V_An_* is the anode liquid volume, Δ*c* is the change of ethanol concentration [45].

### Sample characterization

The concentrations of Fe(II) were measured by the ferrozine assay as previously reported [43]. The production of acetate was monitored by ultra-high-performance liquid chromatography (Thermo U-3000; Thermo Fisher Scientific, Waltham, MA) equipped with an Aminex NPX-87H column (Bio-Rad, Hercules, CA) and a UV–Vis detector. The eluent was 8 mM H_2_SO_4_ running at a rate of 0.6 ml min^−1^ and acetate was detected at 210 nm according to a previous study [46]. The consumption of ethanol was determined by a gas chromatograph (Agilent 7890 A; Agilent Technologies, Santa Clara, CA), which was equipped with a headspace automatic sampler, a HPINNOWAX column (Agilent), and a FID detector as previously reported [46]. The NADH/NAD^+^ ratio was measured using NAD^+^/NADH assay kit (Beyotime, China) following instructions.

### Electron microscopic analyses

Cell cultures (10 μL) from the bioelectrochemical system were directly dropped onto a 400-mesh carbon-coated copper grid and were let to standstill for 5 min at room temperature. The grid was then wiped with filter paper and negatively stained with 2% uranyl acetate for 60 s and wiped again [42]. The ready-made sample was imaged under a transmission electron microscope (TEM) operating at 80 kV (Tecnai T12, ThermoFisher Scientific). For microtome sectioning, co-culture cells were embedded in LR White embedding resin. In particular, to stain cytochromes in the coculture, the cytochrome reactive histochemical stain 3,3′-diaminobenzidine was applied to the co-culture before embedding as previously described [47]. The obtained resin blocks were sectioned at 70 nm with a diamond knife (DiATOME Ultra 35°, Diatome Ltd, Nidau, Switzerland), and floating sections were applied to grids. Thin sections were then viewed under TEM operating at 80 kV.

### Florescence *in situ* hybridization

Florescence *in situ* hybridization (FISH) was conducted as previously described with some modifications [48]. Cell suspensions were collected and fixed in 4% paraformaldehyde at 4°C overnight. After fixation, suspension samples were concentrated and washed with phosphate-buffered saline (PBS, pH 7.2) for three times and resuspended in PBS, then spotted onto Teflon-coated slides and dried at 46°C. Following the dehydration of samples in a gradient of 50, 70, 80, and 95% ethanol for 3 min each and air-dried. Hybridizations with oligonucleotide probes (5′-[CY3]GGCTACCTTGTTACGACTTCACCCCA-3′ for C.i; 5′-[CY5]GAAGACAGGAGGCCCGAAA-3′ for G.s) were performed at 46°C for 3 h with hybridization buffer (20 mM Tris–HCl, 1 M NaCl, 30% formamide, 0.01% SDS, pH 7.2). After hybridization, slides were washed with preheated washing buffer (56 mM NaCl, 20 mM Tris–HCl, 5 mM EDTA, 0.01% SDS, pH 7.2) at 48°C for 0.5 h, and subsequently washed with sterile water and air-dried in dark. Lastly, the FISH samples were applied with an antifade solution (ProLong Diamond, ThermoFisher Scientific), and then were imaged with a confocal laser scanning microscope (Carl Zeiss, Jena, Germany).

## Results

### C.i obtained EET ability in the presence of G.s

**Figure 2:**
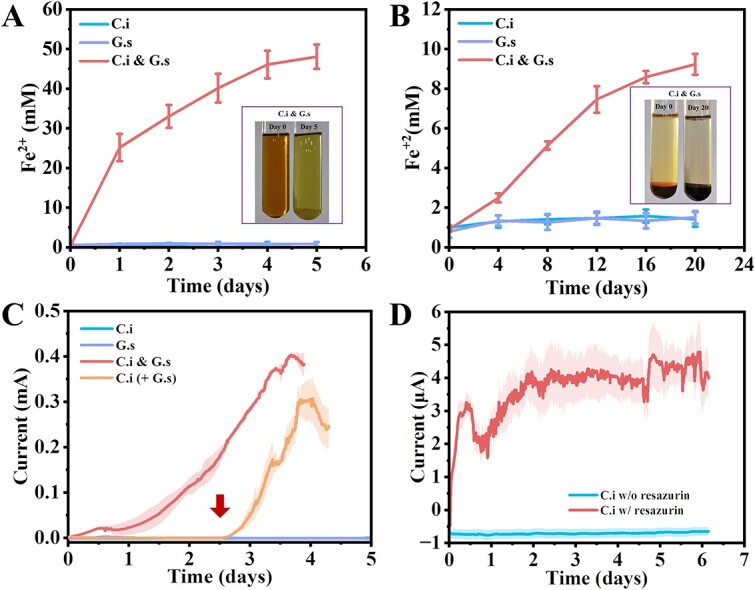
The reduction of extracellular electron acceptors; (A) Fe(III) citrate; (B) ferrihydrite; (C) anode. C.i and G.s were tested; C.i and G.s were inoculated simultaneously (C.i and G.s) or sequentially (C.i + G.s). The arrow indicates the inoculation of G.s; (D) averaged current generation of C.i with and without the supply of 100 μM resazurin; the results shown were the means ± s.d. for quadruple cultures; the shaded area represents one standard deviation.

C.i was predicted to be able to catabolize ethanol since there is an intact ethanol catabolism for an acetate generation pathway encoded on its genome. Here, ethanol will first be catalyzed by the ethanol dehydrogenases (ADHs) to generate acetaldehyde and then to acetate by acetaldehyde dehydrogenases with the concomitant reduction of NAD^+^ to NADH (Fig. 1). However, C.i was not able to ferment ethanol for hydrogen production due to the thermodynamically unfavorable reaction of NADH oxidation for hydrogen generation catalyzed by [FeFe]-hydrogenase. In addition, C.i. alone was not able to oxidize ethanol via reducing extracellular electron acceptors, neither soluble Fe(III) citrate, solid Fe(III) oxide, nor ITO anode (Fig. 2, Fig. S2). The reason might be that C.i was not able to express EET pathways or be electroactive (Fig. S3) to use those extracellular electron acceptors to discharge the cell for NAD^+^ regeneration then achieving intracellular redox balance, and thereafter the metabolism of ethanol was blocked. We speculated that if an EET pathway was provided, C.i would oxidize ethanol. To demonstrate this, electron shuttles were supplied into the pure C.i culture. Two electron shuttles were selected: (1) resazurin, a lipophilic mediator that is able to pass the cell wall and accepts electrons from periplasm or inner membrane [49], and (2) anthraquinone-2,6-disulfonate (AQDS), a hydrophilic mediator that can only accept electrons from the cell surface [50]. As indicated in Fig. S4, the supplement of AQDS was unable to recover the ethanol oxidation. In contrast, the addition of resazurin achieved the oxidation of ethanol (ca. 0.8 mM day^−1^) (Fig. S4) and the anode reduction in C.i with a maximum current generation of ca. 4.7 μA (Fig. 2D). In particular, the addition of resazurin decreased the proportion of NADH/NAD^+^ in contrast to what occurred in the presence of AQDS showing a ratio comparable to C.i without any treatment (Fig. S5A), as well as, the addition of resazurin also increased the intracellular ATP (Fig. S5B), suggesting a process of oxidative phosphorylation. It is not surprise considering there were NADH-quinone oxidoreductases and F-type ATPase coding genes (Fig. S6) in the genome of C.i. All in all, the results demonstrated that only the exogenous lipophilic mediator of resazurin was able to facilitate the EET of C.i to achieve intracellular redox balance for energy generation and contributed to the sustained ethanol oxidation, and suggested that C.i also lacked the electron transfer pathway across the cell wall/membrane.

G.s is an electroactive bacterium [13] that oxidizes acetate, using EET to reduce extracellular electron acceptors directly for energy generation. It is not capable of growth on ethanol [5, 41]. However, when G.s was incubated with C.i, the two species formed a coculture capable of ethanol oxidation with the concomitant reduction of extracellular electron acceptors (Fig. 2). Namely, it appeared that C.i obtained EET ability. Since G.s could not directly contribute to the oxidation of ethanol, we speculated that G.s probably facilitated or was involved in the EET of C.i in the coculture. Such assumption was further strengthened using a bioelectrochemical system inoculated with C.i. As expected, C.i was unable to oxidize ethanol to reduce the anode during the preliminary stage (Fig. 2C), but the addition of G.s enabled ethanol oxidization and anode reduction. Notably, neither the metabolites nor any cell components of G.s contribute to the EET of C.i, since neither the addition of the growth culture medium of G.s nor the supplementation with cell-free lysate of G.s was able to recover the EET of C.i (Fig. S7). This raises the issue of the mechanism involved with the participation of G.s in the apparent EET of C.i.

### C.i and G.s form energetic coupling

**Figure 3:**
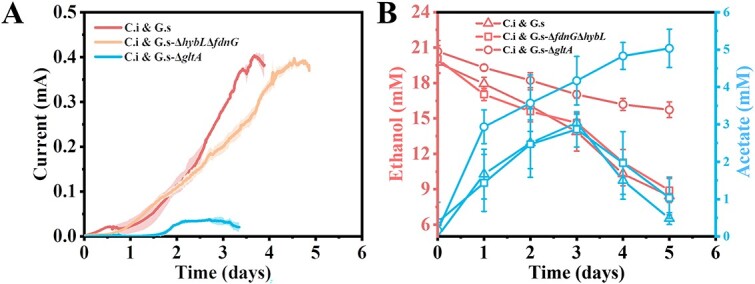
Ethanol oxidation and anode reduction of mixed species coculture; (A) averaged current generation of C.i and wild-type G.s coculture, C.i and G.s-Δ*fdnG*Δ*hybL* (formate dehydrogenase and uptake hydrogenase double deletion strain of G.s) coculture, and C.i and G.s-Δ*gltA* (citrate synthase deletion strain of G.s) coculture; the shaded area represents one standard deviation; four independent tests were performed for each culture; (B) ethanol consumption and acetate accumulation in coculture of C.i and G.s, C.i and G.s-Δ*fdnG*Δ*hybL*, C.i and G.s-Δ*gltA* growing in a bioelectrochemical system; the results shown were the means ± s.d. for quadruple cultures.

Previous studies of other *Clostridium* species showed that the formation of either hydrogen or formate provided a mechanism for recycling the NADH via discharge of the NADH [51–53], and hydrogen and formate could be further oxidized by G.s for extracellular electron acceptors reduction [54, 55]. To identify the possibility of hydrogen or formate facilitating the EET of C.i in coculture, both a formate dehydrogenase and uptake hydrogenase-deficient G.s strain, G.s-Δ*fdnG*Δ*hybL*, which is not able to oxidize formate and hydrogen, was cocultured with C.i in the anode chamber of a bioelectrochemical system. As demonstrated in Fig. 3, the deficiency of formate dehydrogenase and uptake hydrogenase did not affect the ethanol oxidation and the anode reduction of the coculture, and a comparable maximum current of ca. 0.4 mA compared to the wildtype coculture was generated. In addition, neither hydrogen nor formate could be detected in either C.i single culture or the coculture. So, neither hydrogen nor formate was involved in the EET of C.i and a direct interspecies electron transfer (DIET) between these two species was hypothesized.

C.i oxidizes ethanol to generate electrons and acetate (Fig. 3B). If DIET could be established in the coculture, those electrons could be transferred to G.s, to ultimately arrive at the anode. Meanwhile, the acetate could be further oxidized by G.s to contribute to the anode reduction for current generation. So, theoretically, two electron sources of C.i and acetate are projected to contribute to the current generation of G.s if the DIET occurred. To verify this process, a citrate synthase-deficient G.s strain G.s-Δ*gltA*, which was unable to oxidize acetate but could assimilate acetate, was cocultured with C.i. In theory, in this coculture, only the electrons released by C.i would be able to contribute to the current generation and a lower current would be generated. As expected, acetate was accumulated in the anolyte and a much lower current with a maximum of 0.04 mA was generated (Fig. 3A), and a much lower coulombic efficiency of 1.55% was calculated compared to the 31.80% of the wild-type coculture. The decline of current generation in the mutant coculture also suggested that acetate oxidation by G.s primarily contributed to the current generation of the wild-type coculture. Notably, ethanol oxidation was also impaired in this mutant coculture, with only 5 mM ethanol consumed in 5 days compared to 12 mM ethanol consumed in 5 days of the wildtype coculture (Fig. 3B). The reason may due to the accumulation of acetate, which restrained the ethanol oxidation to acetate generation thermodynamically.

Meanwhile, both strain G.s and strain G.s-Δ*gltA* grew alongside C.i and formed biofilms on the anode (Fig. S8). So, it shows a mutualistic symbiosis that G.s conferred the EET to C.i achieving energy metabolism via oxidative phosphorylation and thereafter contributed to the growth of C.i, and in return, the electrons released by C.i were also able to be used by G.s for energy generation supporting the growth of G.s. More precisely, those two species of C.i and G.s formed an intimate energetic coupling to achieve electrosyntrophic growth via DIET. Previous studies indicated that extracellular cytochromes in electron donor bacterial species usually facilitated the DIET and were necessary for the formation of electrosyntrophy with the other electron acceptor bacterial species [41, 56]. However, C.i lacks cytochrome-encoding genes on its genome. The mechanism of electron transfer between those two species remains an enigma.

### Energetic coupling is established after the formation of interspecies cell fusion

**Figure 4:**
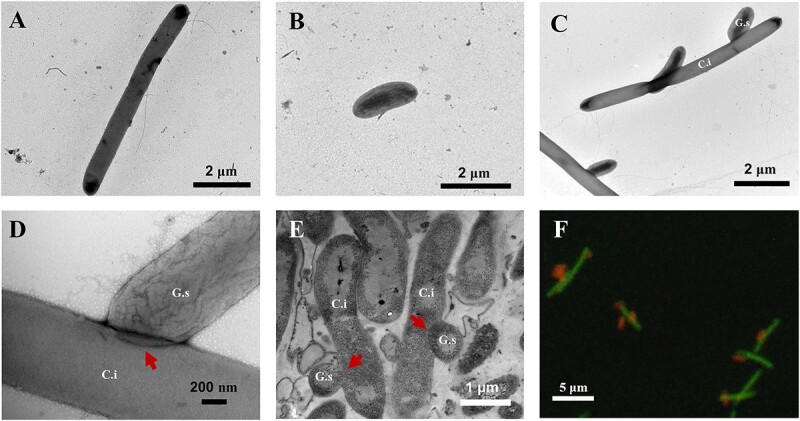
Microscopy of C.i and G.s in pure culture and coculture; transmission electron microscopy image of C.i (A) and G.s (B); (C) transmission electron microscopy image of the C.i and G.s coculture; (D) transmission electron microscopy image of the intersection between C.i and G.s; (E) Image of thin section of coculture cells_;_ arrows indicate the interspecies cell fusion; (F) confocal laser scanning microscope image of the coculture after fluorescence *in situ* hybridization of species-specific probes; the C.i cell was labeled with a green probe, and the G.s cell was labeled with a red probe; coculture cells were collected from the bioelectrochemical system; shown were representative images from at least 10 images.

It was also presumed that the formation of direct physical connection is necessary for the DIET between electron donor and electron acceptor bacterial species, either by direct contact or by forming conductive cell aggregates (Fig. S9A) [5, 41, 57]. However, no visible cell aggregates could be seen directly in the coculture (Fig. S9B). Transmission electron microscopy was performed further to examine the possible physical connection between those two species. The length of a C.i is about 10 μm (Fig. 4A), while the G.s cells have an average length of about 1–2 μm (Fig. 4B), making it easy to distinguish the two species. Figure 4C shows that G.s attached to C.i at the cell pole (Fig. 4C and D) and fused with C.i but kept itself integral by way of cell fusion (probably via the fusion of cell walls) (Fig. 4D and E and Fig. S11). This is in contrast to the direct cell surface contact of the previous reported DIET cocultures [5, 9, 58], and it is reminiscent of previously reported interspecies cell fusion also involving *Clostridium* cells [59]. In addition, some C.i cells had multiple G.s cells fused to them (Fig. S10). The direct contact between C.i and G.s was also verified by FISH after labeling each species with different species-specific fluorescent probes (Fig. 4F).

**Figure 5:**
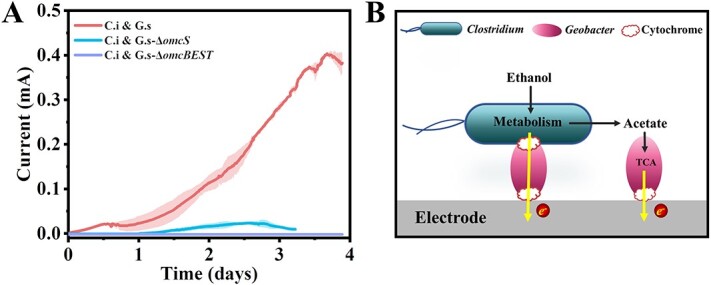
The reduction of extracellular electron acceptors by coculture; (A) anode reduction by cocultures of C.i and G.s, C.i and G.s-Δ*omcS* (strain of G.s-deficient in the expression of extracellular cytochrome OmcS), and C.i and G.s-Δ*omcBEST* (strain of G.s-deficient in the expression of quadruple cytochromes of OmcB, OmcE, OmcS, and OmcT); the shaded area represents one standard deviation; four independent tests were performed for each culture; (B) the extracellular electron acceptor reduction model in C.i and G.s coculture; C.i oxidized ethanol and produced electrons and acetate; those electrons were directly transferred into G.s via the extracellular cytochromes of G.s and were further transferred to extracellular electron acceptors by G.s; meanwhile, the acetate was released into the culture medium which would be oxidized by G.s to reduce extracellular electron acceptors directly.

Cytochromes are necessary for DIET [41, 60]. The distribution of cytochromes in the coculture was examined after staining by the histochemical stain 3,3′-diaminobenzidine, which could be catalyzed by hemes of cytochromes to form oxidized precipitate with a high electron opacity in transmission electron microscopy images in the presence of hydrogen peroxide [47]. As indicated in Fig. S11, only G.s cells were stained dark, indicating that there were abundant cytochromes on G.s but not on C.i. OmcS was thought to be necessary for G.s to directly accept electrons from the electron-donating species in the DIET coculture [5, 41]. To identify the possibility that OmcS facilitated the DIET in the coculture, the *omcS* mutant G.s strain G.s-Δ*omcS* was cocultured with C.i in a bioelectrochemical system and the current generation was monitored. Considering OmcS was also thought to be involved in the anode reduction of G.s [42], the current generation of single G.s-Δ*omcS* was also compared. As shown in Fig. S12, the deletion of *omcS* only partially impaired the current generation of G.s as previously reported [42], with a maximum of ca. 0.23 mA, which is in contrast to the maximum of ca. 0.4 mA in wildtype G.s and the coculture. However, the deletion of *omcS* greatly impaired the current generation of the coculture, only generating a maximum current of ca. 0.02 mA (Fig. 5A). This result indicated that OmcS participated in the interspecies electron transfer in the coculture. We further deleted three other of the most abundant extracellular c-type cytochromes, namely OmcB, OmcE, and OmcT, in strain G.s-Δ*omcS*, generating strain G.s-Δ*omcBEST*, and cocultured this strain with C.i in the bioelectrochemical system. As demonstrated in Fig. 5A, the current generation of this coculture was completely blocked, even though the single G.s-Δ*omcBEST* strain was still able to reduce the anode, generating a maximum current of ca. 0.1 mA (Fig. S12). All of these results indicated that the DIET between C.i and G.s was facilitated by extracellular cytochromes of G.s or cytochromes from G.s conferred the EET on C.i.

## Discussion

The results presented here are consistent with a new EET mechanism for Gram-positive bacteria via the recruitment of the EET pathway from an EET capable Gram-negative bacteria. C.i could not metabolize ethanol since it could not perform EET to discharge NADH, thus achieving intracellular redox balance. In contrast, G.s is able to express abundant extracellular cytochromes to facilitate EET. Our results showed that the pole end of G.s attached on C.i after cell fusion by which the G.s cytochrome dependent EET pathway was integrated with the oxidation of NADH in C.i, allowing the cycling regeneration of NAD^+^ to drive ethanol metabolism and thereby discharging the C.i cell and generating the energy for C.i. We conclude that those electrons should not be transferred to extracellular electron acceptors directly, otherwise they would be transferred into G.s and used by G.s to contribute to the reduction of extracellular electron acceptors for energy generation. In the meantime, acetate was generated during ethanol metabolism. Although G.s could couple the oxidation of acetate with the reduction of extracellular electron acceptors, the direct oxidation of acetate by G.s contributed to the reduction of the majority of extracellular electron acceptors. Ultimately, those two species formed energetic coupling to achieve ethanol oxidation and extracellular electron acceptor, such as anode, reduction cooperatively (Fig. 5B).

The energetic coupling represents a cooperating energy metabolism between C.i and G.s, which is in contrast to the previously reported coculture between *Clostridium pasteurianum* and G.s displaying a substance metabolism interaction [61]. In the coculture, *C. pasteurianum* was able to grow independently via glucose fermentation with the production of acetate. G.s oxidized the acetate to release electrons which were further taken up by *C. pasteurianum* via its transmembrane flavin-bound polyferredoxin and thereafter affected its intracellular redox state. Meanwhile, G.s secreted cobamide molecules that could modify the metabolic pathway of *C. pasteurianum* [37]. So, in the coculture, *C. pasteurianum* showed a metabolic shift with a higher acetate and hydrogen yield. In our coculture, the interspecies electron transfer was coupled with the energy metabolism of both species and the secretion of metabolite did not contribute to the energetic coupling.

Our results suggest a heretofore unknown contribution of cell fusion to energetic coupling. However, the interspecies cell fusion in our coculture seems logical and inevitable, considering the non-conductivity of C.i cell wall. In previous studies, exchange of materials between cells after cell fusion has been shown to be necessary for the formation of interspecies energetic coupling [62, 63]. For example, in the *Clostridium acetobutylicum* and *Desulfovibrio vulgaris* coculture, the exchange of cytoplasmic molecules between those two species contributed to the survival of *D. vulgaris* in its nutrient-deficient environment and the enhancement of hydrogen fermentation of *C. acetobutylicum* [62]. Similarly, in a *Clostridium* coculture of *C. ljungdahlii* and *C. acetobutylicum*, the direct cell fusion allowed the exchange of metabolites that enabled complete carbon utilization and expanded the metabolic product spectrum of individual species [59]. In contrast, the energetic coupling via C.i and G.s cell fusion of this study was based upon direct electron exchange. We did not think the possibility of material exchange, such as the exchange of cytoplasmic reducing power of NAD(P)H or FdH_2_, between C.i and G.s cells, considering that both cells kept intact cell shapes after cell fusion and the extracellular EET pathway of G.s is necessary for the energetic coupling of the coculture. A previous study also suggested that nutritional stress could trigger cell fusion between bacterial species [62]. So, we speculated that the nutritional stress from both C.i and G.s should also trigger the cell fusion of those two species in our study which is warranted to further study.

The formation of DIET between non-electroactive bacteria and electroactive bacteria is different from previously well-characterized classical electronic syntrophic coculture composed by both electroactive microorganisms [31, 41, 56]. In those electrosyntrophic cocultures, cytochromes from the electron donor species are required for the DIET, and the expression of cytochromes in electron donor species acts as predictors to select electrosyntrophic cocultures [9]. Here, we showed that the cytochrome-free *Clostridium* species could utilize cytochromes from the partner electron acceptor species to set up DIET. In addition, a new interspecies connection mode in DIET coculture via cell fusion was suggested. So, the discovery extends the knowledge on DIET. Furthermore, our study also provides a new perspective on the understanding of ectosymbiosis i.e. a symbiont dwelling on the external body of the host, which has never been reported between bacterial species. Notably, even though G.s invaded the cell wall of C.i, the relationship between those two species should also be mutualism, considering that the survival of any species is dependent on the metabolism of its partner species in the coculture.

Our study also provides a perspective that may lead to a better understanding of the ecology of microbial communities. In general, *Clostridium* performs fermentation to generate energy for cell growth [64]. So, a low energy production mechanism of substrate level phosphorylation was involved in the ATP generation. *Clostridium* also has NADH quinone oxidoreductase and ATPase. By forming energetic coupling with electroactive *Geobacter* species, *Clostridium* activated the NADH quinone oxidoreductase (Fig. S13) and performed energy-efficient oxidative phosphorylation via EET to flare off electrons on extracellular electron acceptors. Here, more energy could be harvested and the survival of *Clostridium* in microbial communities should be favorable. Actually, previous studies demonstrated that *Clostridium* species are widely present in mixed-culture BESs and there is a positive correlation between the dominance of *Clostridium* and current generation [35, 65]. Correspondingly, a “division-of-labor” model to explain the interspecific relationship in those mixed species electroactive microbial communities was suggested [66, 67]. In this model, fermentative bacteria such as *Clostridium* species ferment complex organics to produce small molecular organic acids, which further are able to act as electron donors to be oxidized by electroactive bacteria for the current generation. However, this is not contrary to our electrosyntrophic coculture model since the majority of electroactive *Geobacter* still lived on the catabolite of *Clostridium*, and our study illustrated another possible interaction of direct electronic connection between those two species.

It is striking that *Clostridium* received a new mode of energy metabolism after capturing *Geobacter* cells, which is reminiscent of the evolution of the mitochondrion [68]. The mitochondrion is generally recognized as an exogenously evolved organelle functioning as a specialized energy generation machine for the host cell by which a symbiotic relationship via energetic coupling was established. Even though the origin of mitochondrion is controversial, the recent data indicated that mitochondrion originated from endosymbiosis of bacteria in archaeal host cell. However, geologic evidences and genetic analyses of archaea exclude an endocytic event to contribute to this intracellular symbiosis [69, 70], and the formation of mitochondrion is mysterious. In contrast, our study showed that *Geobacter* cells contributed to a new energy metabolism of *Clostridium* cells after attached on and only partially fused with the host. This leads us to the speculation that interspecies cell fusion could be the mechanism by which archaea acquired new abilities and became pre-mitochondria; partial fusion between bacterial species may have played an important role in the genesis and evolution of mitochondria.

As a final note, we point out that the single non-electroactive *Clostridium* achieved electroactivity after coculture with electroactive *Geobacter*, a result that suggest that we may need a more expansive definition of electroactive bacteria. To this end, a new biotechnology to generate electroactive bacteria via synthetic microbial communities rather than the complex synthetic biology is not irrational. In addition, these findings also imply more functional diversity of bacteria in a community than the single cell, highlighting the importance of studying microorganisms at the community level. Finally, our study also provides an explanation for the persistence of unculturable bacteria and has profound implications for ecological or community engineering of microbial ecosystems.

## Conflicts of interest

The authors declare no competing financial interests.

## Funding

This work was supported by the National Science Fund for Distinguished Young Scholars of China, Grant No. 41925028, National Science Fund for Excellent Young Scholars of China, Grant No. 42222703, and National Natural Science Foundation of China, Grants No. 92251301 and 42077218.

## Data availability

All data generated or analysed during this study are included in this published article [and its supplementary information files].

## Supplementary Material

ISME_Supplementary_figures_ycae058

## References

[1] Garbini GL , Barra Caracciolo A, Grenni P. Electroactive bacteria in natural ecosystems and their applications in microbial fuel cells for bioremediation: a review. Microorganism*s* 2023;11:11. 10.3390/microorganisms11051255 PMC10263229 37317229

[2] Myers CR , Nealson KH. Bacterial manganese reduction and growth with manganese oxide as the sole electron acceptor. Scienc*e* 1988;240:1319–21. 10.1126/science.240.4857.1319 17815852

[3] Lovley DR , Stolz JF, Nord GL et al. Anaerobic production of magnetite by a dissimilatory iron-reducing microorganism. Natur*e* 1987;330:252–4. 10.1038/330252a0

[4] Bond DR , Holmes DE, Tender LM et al. Electrode-reducing microorganisms that harvest energy from marine sediments. Scienc*e* 2002;295:483–5. 10.1126/science.1066771 11799240

[5] Summers ZM , Fogarty HE, Leang C et al. Direct exchange of electrons within aggregates of an evolved syntrophic coculture of anaerobic bacteria. Scienc*e* 2010;330:1413–5. 10.1126/science.1196526 21127257

[6] Kato S , Hashimoto K, Watanabe K. Microbial interspecies electron transfer via electric currents through conductive minerals. Proc Natl Acad Sci US*A* 2012;109:10042–6. 10.1073/pnas.1117592109 22665802 PMC3382511

[7] Lovley DR . Electrotrophy: other microbial species, iron, and electrodes as electron donors for microbial respirations. Bioresour Techno*l* 2022;345:126553. 10.1016/j.biortech.2021.126553 34906705

[8] Lovley DR . Syntrophy goes electric: direct interspecies electron transfer. Ann Rev Microbio*l* 2017;71:643–64. 10.1146/annurev-micro-030117-020420 28697668

[9] McGlynn SE , Chadwick GL, Kempes CP et al. Single cell activity reveals direct electron transfer in methanotrophic consortia. Natur*e* 2015;526:531–5. 10.1038/nature15512 26375009

[10] Ha PT , Lindemann SR, Shi L et al. Syntrophic anaerobic photosynthesis via direct interspecies electron transfer. Nat Commu*n* 2017;8:13924. 10.1038/ncomms13924 28067226 PMC5227917

[11] Liu X , Huang L, Rensing C et al. Syntrophic interspecies electron transfer drives carbon fixation and growth by *Rhodopseudomonas palustris* under dark, anoxic conditions. Sci Ad*v* 2021;7:eabh1852. 10.1126/sciadv.abh1852 PMC11057707 34215588

[12] Yang WH , Weber KA, Silver WL. Nitrogen loss from soil through anaerobic ammonium oxidation coupled to iron reduction. Nat Geosc*i* 2012;5:538–41. 10.1038/ngeo1530

[13] Logan BE , Rossi R, Ragab A et al. Electroactive microorganisms in bioelectrochemical systems. Nat Rev Microbio*l* 2019;17:307–19. 10.1038/s41579-019-0173-x 30846876

[14] Lovley DR , Holmes DE. Electromicrobiology: the ecophysiology of phylogenetically diverse electroactive microorganisms. Nat Rev Microbio*l* 2022;20:5–19. 10.1038/s41579-021-00597-6 34316046

[15] Shi L , Dong H, Reguera G et al. Extracellular electron transfer mechanisms between microorganisms and minerals. Nat Rev Microbio*l* 2016;14:651–62. 10.1038/nrmicro.2016.93 27573579

[16] Zhao J , Li F, Cao Y et al. Microbial extracellular electron transfer and strategies for engineering electroactive microorganisms. Biotechnol Ad*v* 2021;53:107682. 10.1016/j.biotechadv.2020.107682 33326817

[17] Ueki T . Cytochromes in extracellular electron transfer in *Geobacter*. Appl Environ Microbio*l* 2021;87:e03109–20 33741623 10.1128/AEM.03109-20 PMC8117768

[18] Wang F , Craig L, Liu X et al. Microbial nanowires: type IV pili or cytochrome filaments? Trends Microbio*l* 2023;31:384–92. 10.1016/j.tim.2022.11.004 36446702 PMC10033339

[19] Saffarini D , Brockman K, Beliaev A et al. *Shewanella oneidensis* and extracellular electron transfer to metal oxides. In: Saffarini D (ed.), Bacteria-Metal Interactions. Cham: Springer International Publishing, 2015, 21–40, 10.1007/978-3-319-18570-5_2.

[20] Marsili E , Baron DB, Shikhare ID et al. *Shewanella* secretes flavins that mediate extracellular electron transfer. Proc Natl Acad Sci US*A* 2008;105:3968–73. 10.1073/pnas.0710525105 18316736 PMC2268775

[21] Roden EE , Kappler A, Bauer I et al. Extracellular electron transfer through microbial reduction of solid-phase humic substances. Nat Geosc*i* 2010;3:417–21. 10.1038/ngeo870

[22] Piepenbrock A , Kappler A. Humic substances and extracellular electron transfer. In: Gescher J., Kappler A. (eds.), Microbial Metal Respiration: From Geochemistry to Potential Application*s*. Berlin Heidelberg: Springer, 2012, 107–28

[23] Newman DK , Kolter R. A role for excreted quinones in extracellular electron transfer. Natur*e* 2000;405:94–7. 10.1038/35011098 10811225

[24] Hernandez ME , Kappler A, Newman DK. Phenazines and other redox-active antibiotics promote microbial mineral reduction. Appl Environ Microbio*l* 2004;70:921–8. 10.1128/AEM.70.2.921-928.2004 14766572 PMC348881

[25] Rohde M . The gram-positive bacterial cell wall. Microbiol Spect*r* 2019;7. 10.1128/microbiolspec.GPP3-0044-2018 PMC11086966 31124431

[26] Pasquina-Lemonche L , Burns J, Turner RD et al. The architecture of the gram-positive bacterial cell wall. Natur*e* 2020;582:294–7. 10.1038/s41586-020-2236-6 32523118 PMC7308169

[27] Freguia S , Masuda M, Tsujimura S et al. *Lactococcus lactis* catalyses electricity generation at microbial fuel cell anodes via excretion of a soluble quinone. Bioelectrochemistr*y* 2009;76:14–8. 10.1016/j.bioelechem.2009.04.001 19411192

[28] Light SH , Su L, Rivera-Lugo R et al. A flavin-based extracellular electron transfer mechanism in diverse gram-positive bacteria. Natur*e* 2018;562:140–4. 10.1038/s41586-018-0498-z 30209391 PMC6221200

[29] Yang Y , Wang Z, Gan C et al. Long-distance electron transfer in a filamentous gram-positive bacterium. Nat Commu*n* 2021;12:1709. 10.1038/s41467-021-21709-z 33731718 PMC7969598

[30] Carlson HK , Iavarone AT, Gorur A et al. Surface multiheme c-type cytochromes from *Thermincola potens* and implications for respiratory metal reduction by gram-positive bacteria. Proc Natl Acad Sci US*A* 2012;109:1702–7. 10.1073/pnas.1112905109 22307634 PMC3277152

[31] Gavrilov SN , Zavarzina DG, Elizarov IM et al. Novel extracellular electron transfer channels in a gram-positive thermophilic bacterium. Front Microbio*l* 2021;11:11. 10.3389/fmicb.2020.597818 PMC7829351 33505370

[32] Joseph RC , Kim NM, Sandoval NR. Recent developments of the synthetic biology toolkit for *clostridium*. Front Microbio*l* 2018;9:154. 10.3389/fmicb.2018.00154 29483900 PMC5816073

[33] Luo H , Zheng P, Xie F et al. Co-production of solvents and organic acids in butanol fermentation by *Clostridium acetobutylicum* in the presence of lignin-derived phenolics. RSC Ad*v* 2019;9:6919–27. 10.1039/C9RA00325H 35518483 PMC9061099

[34] Pham TH , Boon N, Aelterman P et al. Metabolites produced by *Pseudomonas* sp. enable a gram-positive bacterium to achieve extracellular electron transfer. Appl Microbiol Biotechno*l* 2008;77:1119–29. 10.1007/s00253-007-1248-6 17968538

[35] Kracke F , Vassilev I, Kromer JO. Microbial electron transport and energy conservation - the foundation for optimizing bioelectrochemical systems. Front Microbio*l* 2015;6:575. 10.3389/fmicb.2015.00575 26124754 PMC4463002

[36] Schwab L , Rago L, Koch C et al. Identification of *Clostridium cochlearium* as an electroactive microorganism from the mouse gut microbiome. Bioelectrochemistr*y* 2019;130:107334. 10.1016/j.bioelechem.2019.107334 31352302

[37] Berthomieu R , Pérez-Bernal MF, Santa-Catalina G et al. Mechanisms underlying *Clostridium pasteurianum*'s metabolic shift when grown with *Geobacter sulfurreducens*. Appl Microbiol Biotechno*l* 2022;106:865–76. 10.1007/s00253-021-11736-7 34939136

[38] Boto ST , Bardl B, Harnisch F et al. Microbial electrosynthesis with *Clostridium ljungdahlii* benefits from hydrogen electron mediation and permits a greater variety of products. Green Che*m* 2023;25:4375–86. 10.1039/D3GC00471F 37288452 PMC10243432

[39] Kiely PD , Regan JM, Logan BE. The electric picnic: synergistic requirements for exoelectrogenic microbial communities. Curr Opin Biotechno*l* 2011;22:378–85. 10.1016/j.copbio.2011.03.003 21441020

[40] dos Passos VF , Marcilio R, Aquino-Neto S et al. Hydrogen and electrical energy co-generation by a cooperative fermentation system comprising clostridium and microbial fuel cell inoculated with port drainage sediment. Bioresour Techno*l* 2019;277:94–103. 10.1016/j.biortech.2019.01.031 30660066

[41] Liu X , Zhuo S, Rensing C et al. Syntrophic growth with direct interspecies electron transfer between pili-free *Geobacter* species. ISME *J* 2018;12:2142–51. 10.1038/s41396-018-0193-y 29875437 PMC6092431

[42] Ye Y , Liu X, Nealson Kenneth H et al. Dissecting the structural and conductive functions of nanowires in *Geobacter sulfurreducens* electroactive biofilms. mBi*o* 2022;13:e03822–1. 10.1128/mbio.03822-21 PMC8844916 35164556

[43] Liu X , Ye Y, Xiao K et al. Molecular evidence for the adaptive evolution of *Geobacter sulfurreducens* to perform dissimilatory iron reduction in natural environments. Mol Microbio*l* 2020;113:783–93. 10.1111/mmi.14443 31872462

[44] Liu X , Ye Y, Zhang Z et al. Prophage induction causes *Geobacter* electroactive biofilm decay. Environ Sci Techno*l* 2023;57:6196–204. 10.1021/acs.est.2c08443 36997849

[45] Liu H , Cheng S, Logan BE. Production of electricity from acetate or butyrate using a single-chamber microbial fuel cell. Environ Sci Techno*l* 2005;39:658–62. 10.1021/es048927c 15707069

[46] Huang L , Liu X, Ye Y et al. Evidence for the coexistence of direct and riboflavin-mediated interspecies electron transfer in *Geobacter* co-culture. Environ Microbio*l* 2020;22:243–54. 10.1111/1462-2920.14842 31657092

[47] Liu X , Zhuo S, Jing X et al. Flagella act as *Geobacter* biofilm scaffolds to stabilize biofilm and facilitate extracellular electron transfer. Biosens Bioelectro*n* 2019;146:111748. 10.1016/j.bios.2019.111748 31586764

[48] Hu L , Huang J, Li H et al. Design, optimization and verification of 16S rRNA oligonucleotide probes of fluorescence in-situ hybridization for targeting *Clostridium* spp. and *Clostridium kluyveri*. J Microbiol Biotechno*l* 2018;28:1823–33. 10.4014/jmb.1805.04057 30301324

[49] Wu Y , Zhu X, Wang X et al. A new electron shuttling pathway mediated by lipophilic phenoxazine via the interaction with periplasmic and inner membrane proteins of *Shewanella oneidensis* MR-1. Environ Sci Techno*l* 2023;57:2636–46. 10.1021/acs.est.2c07862 36652548

[50] Smith JA , Nevin KP, Lovley DR. Syntrophic growth via quinone-mediated interspecies electron transfer. Front Microbio*l* 2015;6:6. 10.3389/fmicb.2015.00121 25741332 PMC4330893

[51] Litti YV , Khuraseva ND, Vishnyakova AV et al. Comparative study on biohydrogen production by newly isolated *Clostridium butyricum* SP4 and *Clostridium beijerinckii* SP6. *Int J Hydrog* Energ*y* 2023;48:27540–56. 10.1016/j.ijhydene.2023.03.424

[52] Sparling R , Islam R, Cicek N et al. Formate synthesis by *Clostridium thermocellum* during anaerobic fermentation. Can J Microbio*l* 2006;52:681–8. 10.1139/w06-021 16917525

[53] Levin DB , Islam R, Cicek N et al. Hydrogen production by *Clostridium thermocellum* 27405 from cellulosic biomass substrates. Int J Hydrog Energ*y* 2006;31:1496–503. 10.1016/j.ijhydene.2006.06.015

[54] Caccavo F Jr , Lonergan DJ, Lovley DR et al. *Geobacter sulfurreducens* sp. nov., a hydrogen- and acetate-oxidizing dissimilatory metal-reducing microorganism. Appl Environ Microbio*l* 1994;60:3752–9. 10.1128/aem.60.10.3752-3759.1994 7527204 PMC201883

[55] Mollaei M , Timmers PHA, Suarez-Diez M et al. Comparative proteomics of *Geobacter sulfurreducens* PCA(T) in response to acetate, formate and/or hydrogen as electron donor. Environ Microbio*l* 2021;23:299–315. 10.1111/1462-2920.15311 33185968 PMC7894505

[56] Rotaru A-E , Shrestha PM, Liu F et al. Direct interspecies electron transfer between *Geobacter metallireducens* and *Methanosarcina barkeri*. Appl Environ Microbio*l* 2014;80:4599–605. 10.1128/AEM.00895-14 24837373 PMC4148795

[57] Liu X , Zhan J, Liu L et al. In situ spectroelectrochemical characterization reveals cytochrome-mediated electric syntrophy in *Geobacter* coculture. Environ Sci Techno*l* 2021;55:10142–51. 10.1021/acs.est.1c00356 34196176

[58] Wegener G , Krukenberg V, Riedel D et al. Intercellular wiring enables electron transfer between methanotrophic archaea and bacteria. Natur*e* 2015;526:587–90. 10.1038/nature15733 26490622

[59] Charubin K , Modla S, Caplan JL et al. Interspecies microbial fusion and large-scale exchange of cytoplasmic proteins and RNA in a syntrophic *clostridium* coculture. mBi*o* 2020;11:11. 10.1128/mBio.02030-20 PMC7468208 32873766

[60] Baquero DP , Cvirkaite-Krupovic V, Hu SS et al. Extracellular cytochrome nanowires appear to be ubiquitous in prokaryotes. Cel*l* 2023;186:2853–2864.e8. 10.1016/j.cell.2023.05.012 37290436 PMC10330847

[61] Zhang Y , Zheng S, Hao Q et al. Respiratory electrogen *Geobacter* boosts hydrogen production efficiency of fermentative electrotroph *Clostridium pasteurianum*. Chem Eng *J* 2023;456:141069. 10.1016/j.cej.2022.141069

[62] Benomar S , Ranava D, Cardenas ML et al. Nutritional stress induces exchange of cell material and energetic coupling between bacterial species. Nat Commu*n* 2015;6:6283. 10.1038/ncomms7283 25704114

[63] Ranava D , Backes C, Karthikeyan G et al. Metabolic exchange and energetic coupling between nutritionally stressed bacterial species: role of quorum-sensing molecules. mBi*o* 2021;12:e02758–20. 10.1128/mBio.02758-20 PMC7845633 33468690

[64] Wang J , Yin Y. *Clostridium* species for fermentative hydrogen production: an overview. Int J Hydrogen Ener*g* 2021;46:34599–625. 10.1016/j.ijhydene.2021.08.052

[65] Rivalland C , Radouani F, Gonzalez-Rizzo S et al. Enrichment of *Clostridia* enhances *Geobacter* population and electron harvesting in a complex electroactive biofilm. Bioelectrochemistr*y* 2022;143:107954. 10.1016/j.bioelechem.2021.107954 34624726

[66] Rafieenia R , Atkinson E, Ledesma-Amaro R. Division of labor for substrate utilization in natural and synthetic microbial communities. Curr Opin Biotechno*l* 2022;75:102706. 10.1016/j.copbio.2022.102706 35255422

[67] Liu Y , Ding M, Ling W et al. A three-species microbial consortium for power generation. Energy Environ Sc*i* 2017;10:1600–9. 10.1039/C6EE03705D

[68] Roger AJ , Muñoz-Gómez SA, Kamikawa R. The origin and diversification of mitochondria. Curr Bio*l* 2017;27:R1177–92. 10.1016/j.cub.2017.09.015 29112874

[69] Burns JA , Pittis AA, Kim E. Gene-based predictive models of trophic modes suggest Asgard archaea are not phagocytotic. Nat Ecol Evo*l* 2018;2:697–704. 10.1038/s41559-018-0477-7 29459706

[70] Mills DB . The origin of phagocytosis in earth history. Interface Focu*s* 2020;10:20200019. 10.1098/rsfs.2020.0019 32642057 PMC7333901

