# Supplementary material for: Nonelectroactive clostridium obtains extracellular electron transfer-capability after forming chimera with Geobacter
Source: ISME Commun. 2024 Apr 25;4(1):ycae058. doi: 10.1093/ismeco/ycae058 (PMC11104457; doi:10.1093/ismeco/ycae058)
Supplement: ISME_Supplementary_figures_ycae058 [file isme_supplementary_figures_ycae058.pdf]

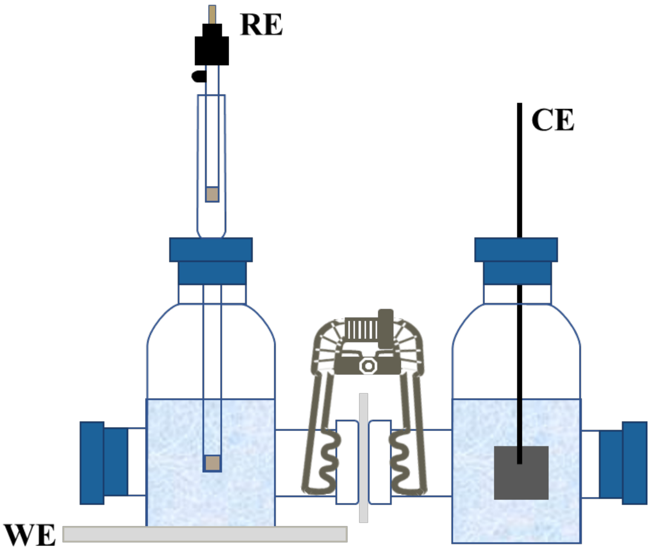


Figure S1. The schematic diagram of the three-electrode H-shaped anaerobic bioelectrochemical system. WE represents Working Electrode. RE represents Reference Electrode. CE represents Counter Electrode.

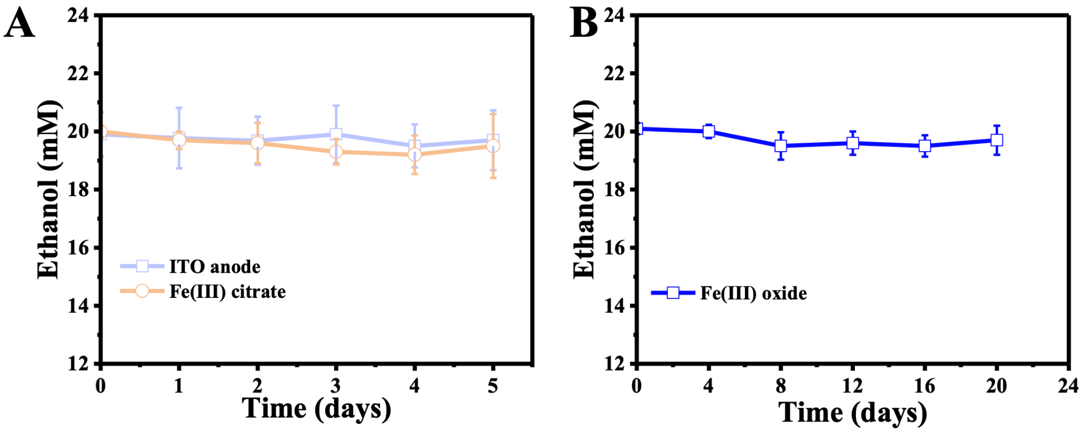


Figure S2. Ethanol metabolism of *C. intestinale* in the presence of different extracellular electron acceptors. (A) Indium tin oxide (ITO) poised at 0.3 V (versus Hg/HgCl sat. KCl) and ferric citrate, and (B) amorphous iron oxide were acted as electron acceptors.

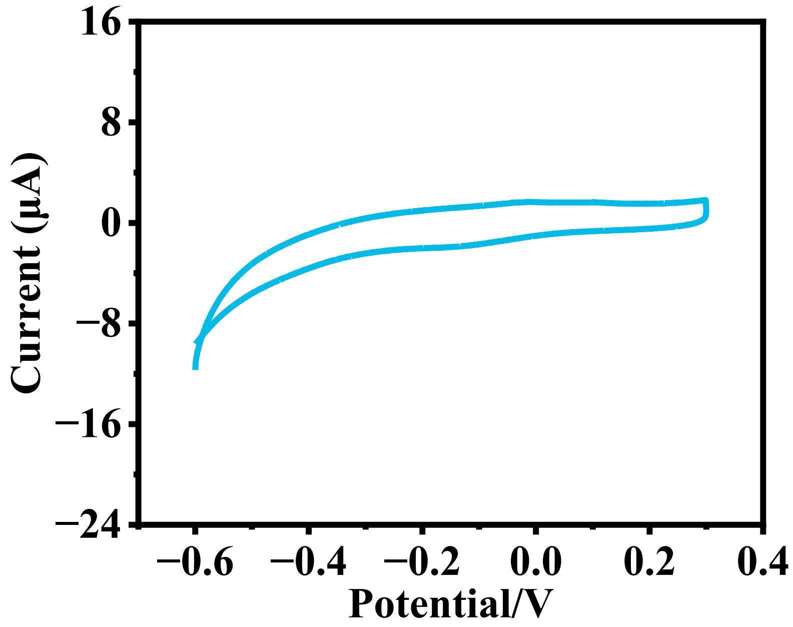


Figure S3. Cyclic voltammogram of *C. intestinale* at the scan rate of 10 mV/s.

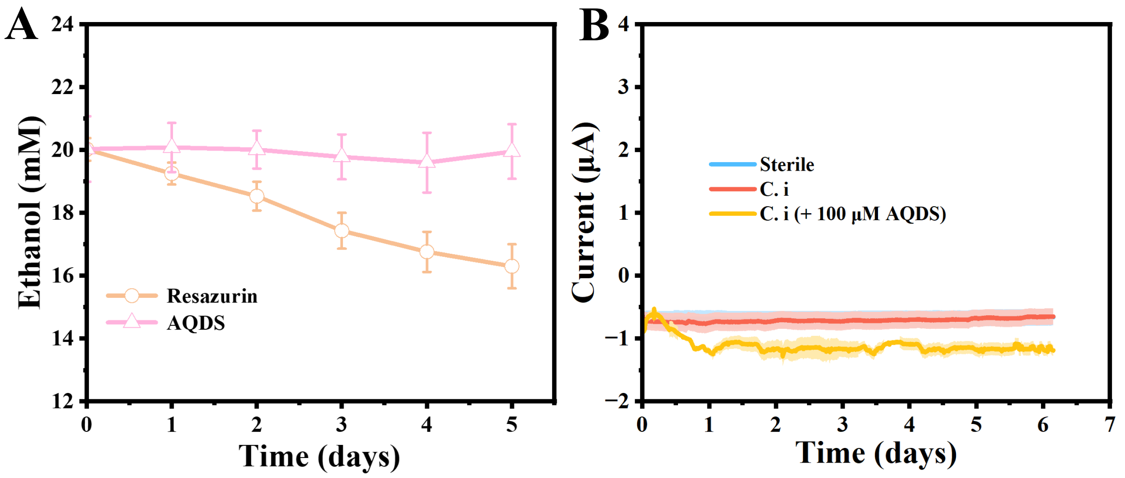


Figure S4. Metabolic characterization of *C. intestinale*. (A) Ethanol metabolism and (B) Current generation. When required, 100 μM AQDS or resazurin was supplied. The shaded area represents one standard deviation. Four independent tests were performed for each treatment.

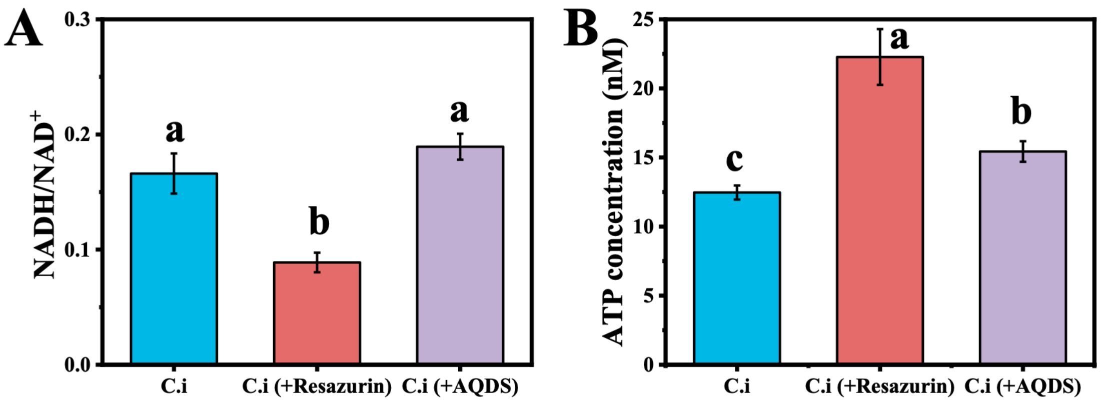


Figure S5. Energy production analysis. The ratio of NADH to $NAD^+$ (A) and the amount of ATP (B) in *C. intestinale*. 100 µM Resazurin or 100 µM AQDS was added when indicated. Columns with different letters are statistically different (LSD test, P<0.05).

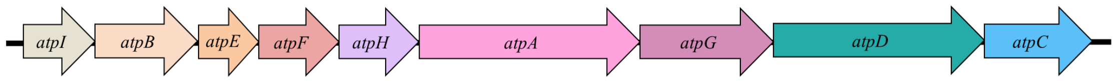


Figure S6. Organization of F-type ATPase synthesis region in *C. intestinale*. The region corresponds to the genome coordinates of *C. intestinale* from 3903746 to 3910443 (Genebank accession number: CP059378.1).

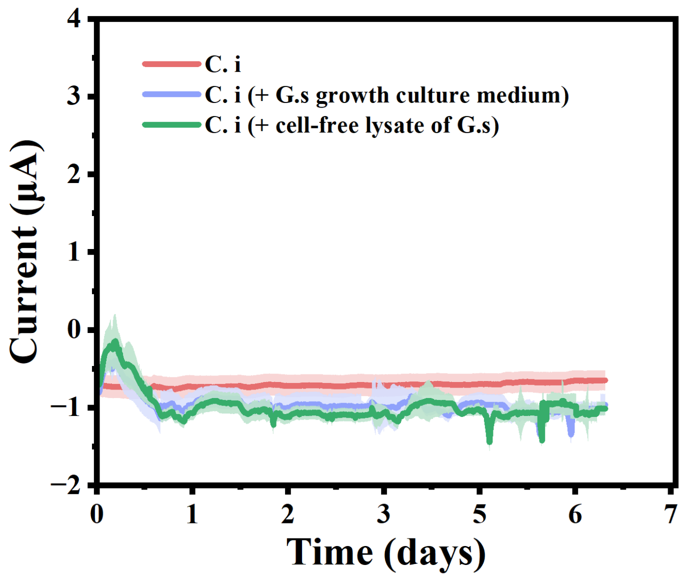


Figure S7. Current generation of *C. intestinale.* When required, 2 mL of cell-free *G. sulfurreducens* growth culture medium or cell-free lysate of 2 mL *G. sulfurreducens* (OD600 = 0.4) was supplied. The shaded area represents one standard deviation. Four independent tests were performed for each treatment.

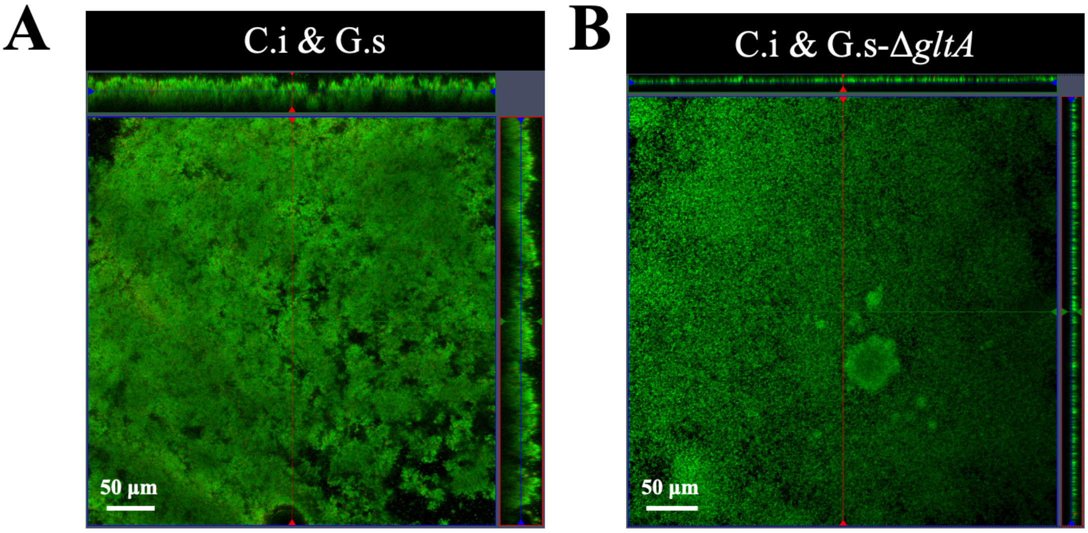


Figure S8. Confocal laser scanning microscopy images of the coculture on the anode. (A) *C. intestinale* and *G. sulfurreducens* (C.i & G.s) coculture. (B) *C. intestinale* and *G. sulfurreducens* mutant deficient in citrate synthase (C.i & G.s-Δ*gltA*) coculture.

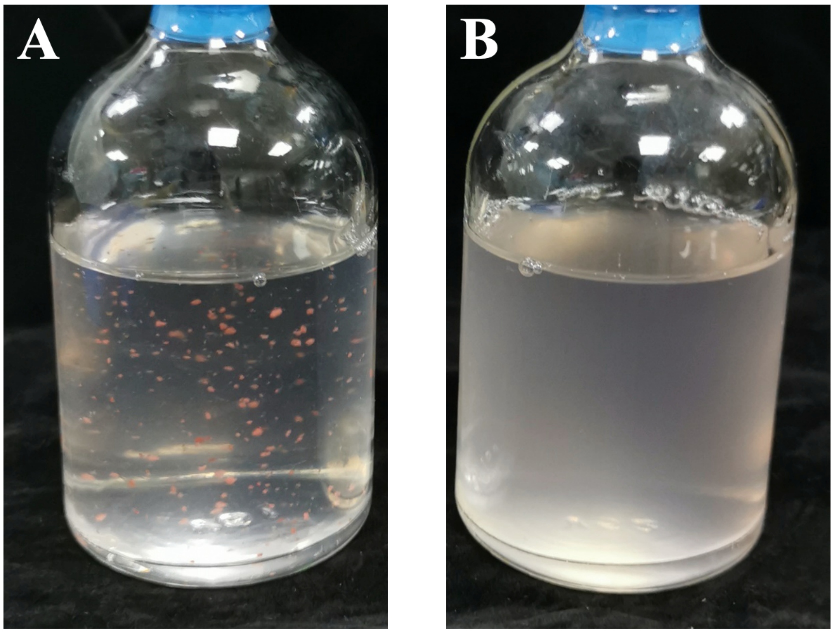


Figure S9. Photographs of bacterial cocultures. (A) Direct interspecies electron transfer coculture between *Geobacter metallireducens* and *Geobacter sulfurreducens*. Cells formed visible aggregates. (B) *C. intestinale* and *G. sulfurreducens* coculture.

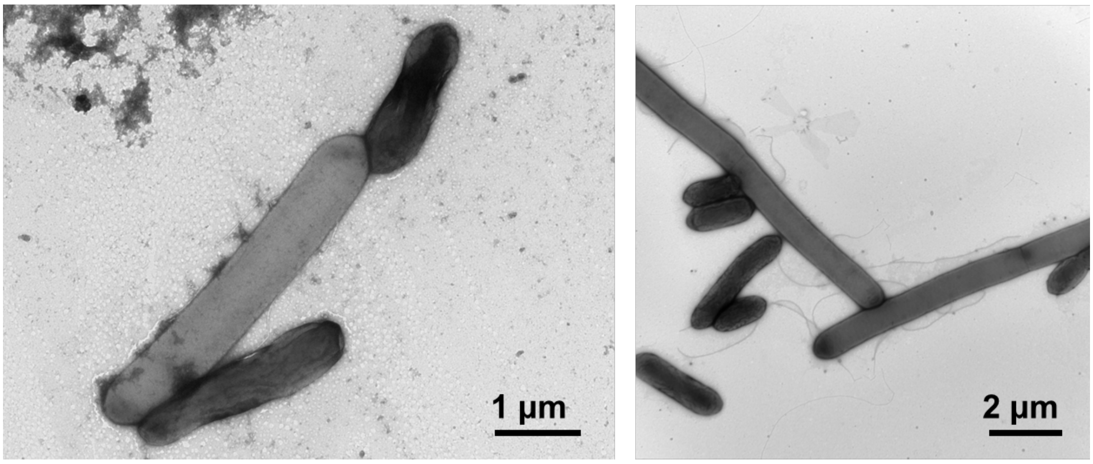


Figure S10. Transmission electron micrographs of *C. intestinale* and *G. sulfurreducens* coculture.

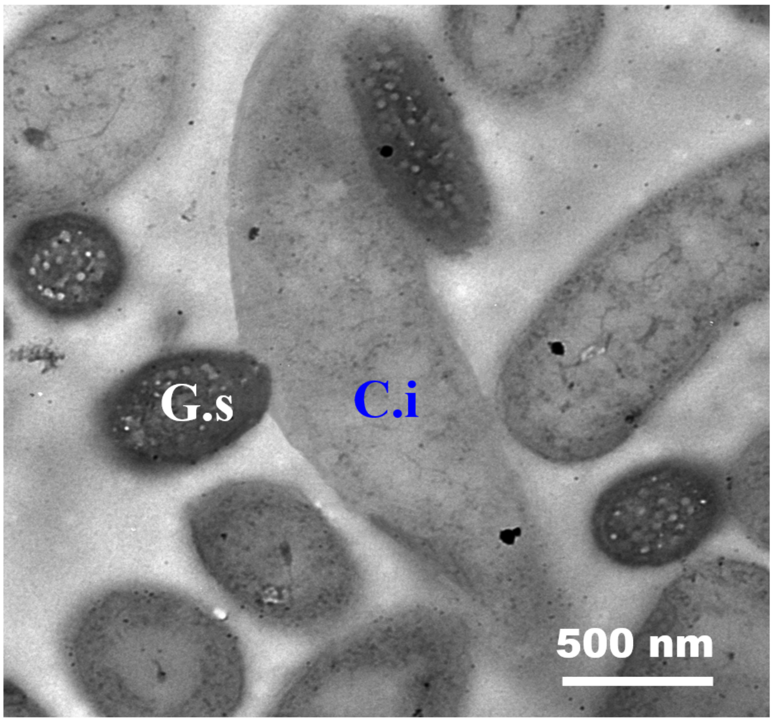


Figure S11. Representative transmission electron micrograph of thin section of coculture cells treated sequentially with DAB and $H_2O_2$.

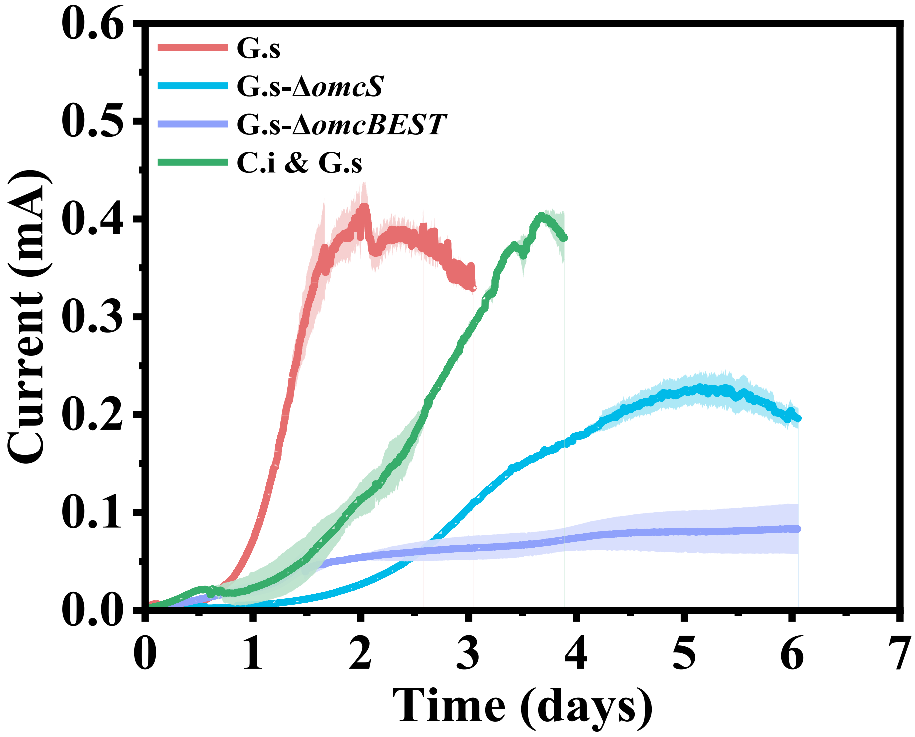


Figure S12. Current generation by *G. sulfurreducens* (G.s), *G. sulfurreducens* extracellular cytochrome OmcS deletion strain (G.s-Δ*omcS*), *G. sulfurreducens* extracellular cytochrome OmcB, OmcE, OmcS, OmcT quadruple deletion strain (G.s-Δ*omcBEST*), and *C. intestinale* and *G. sulfurreducens* coculture (C.i & G.s). Acetate was used as electron donor for tests on all *G. sulfurreducens* single strains, while ethanol was used as electron donor for the coculture. The shaded area represents one standard deviation. At least four independent tests were performed for each culture.

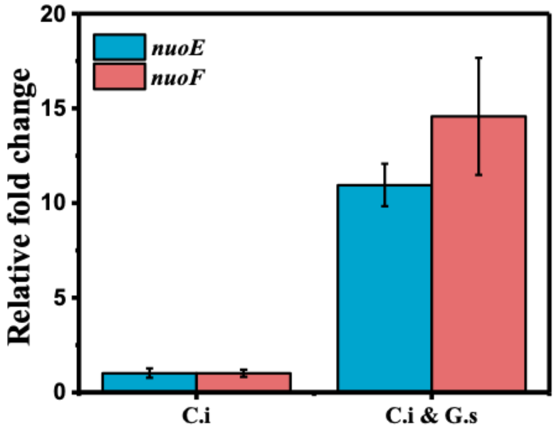


Figure S13. QPCR analyses of the expression of NADH quinone oxidoreductase. *C. intestinale* cells from fermentation growth and coculture growth were compared.
